# Microclimatic Performance of a Free-Air Warming and CO_2_ Enrichment Experiment in Windy Wyoming, USA

**DOI:** 10.1371/journal.pone.0116834

**Published:** 2015-02-06

**Authors:** Daniel LeCain, David Smith, Jack Morgan, Bruce A. Kimball, Elise Pendall, Franco Miglietta

**Affiliations:** 1 United States Department of Agriculture, Agricultural Research Service, Rangelands Resources Research Unit, Northern Plains Area, Fort Collins, CO, United States of America; 2 United States Arid-Land Agricultural Research Center, USDA-ARS, Maricopa, Arizona, United States of America; 3 Hawkesbury Institute for the Environment, University of Western Sydney, Sydney, Australia; 4 Istituto di Biometeorologia, via Giovanni Caproni 8, Firenze, Italy; Chinese Academy of Sciences, CHINA

## Abstract

In order to plan for global changing climate experiments are being conducted in many countries, but few have monitored the effects of the climate change treatments (warming, elevated CO_2_) on the experimental plot microclimate. During three years of an eight year study with year-round feedback-controlled infra-red heater warming (1.5/3.0°C day/night) and growing season free-air CO_2_ enrichment (600 ppm) in the mixed-grass prairie of Wyoming, USA, we monitored soil, leaf, canopy-air, above-canopy-air temperatures and relative humidity of control and treated experimental plots and evaluated ecologically important temperature differentials. Leaves were warmed somewhat less than the target settings (1.1 & 1.5°C day/night) but soil was warmed more creating an average that matched the target settings extremely well both during the day and night plus the summer and winter. The site typically has about 50% bare or litter covered soil, therefore soil heat transfer is more critical than in dense canopy ecosystems. The Wyoming site commonly has strong winds (5 ms^-1^ average) and significant daily and seasonal temperature fluctuations (as much as 30°C daily) but the warming system was nearly always able to maintain the set temperatures regardless of abiotic variation. The within canopy-air was only slightly warmed and above canopy-air was not warmed by the system, therefore convective warming was minor. Elevated CO_2_ had no direct effect nor interaction with the warming treatment on microclimate. Relative humidity within the plant canopy was only slightly reduced by warming. Soil water content was reduced by warming but increased by elevated CO_2_. This study demonstrates the importance of monitoring the microclimate in manipulative field global change experiments so that critical physiological and ecological conclusions can be determined. Highly variable energy demand fluctuations showed that passive IR heater warming systems will not maintain desired warming for much of the time.

## Introduction

Since the industrial revolution, global temperature has increased about 1.0°C due to emission of greenhouse gases. Global temperature is predicted to increase 0.2°C or more per decade at current scenarios of fossil fuel emissions, resulting in a temperature increase of 2.0°C or more by the end of this century [1]. In an attempt to understand and prepare for effects in natural and agronomic ecosystems, researchers are conducting elevated CO_2_ and warming experiments. Additional, long-term field manipulations in natural ecosystems with multiple climate change drivers are needed in order to reduce uncertainties associated with climate—land surface feedbacks [2].

Several methods have been implemented in attempting to simulate atmospheric warming, including use of mini-greenhouse “chambers”, soil heating cables, infrared reflectors and infrared (IR) heaters. A thorough review of these methods and their strengths and weaknesses has been reported [3], as well as each systems ability to warm comparably to actual global warming. Warming with a feedback controlled system of IR heaters is considered to be the superior method [3, 4, 5, 6] because IR heaters do not directly warm the air but warm the plants and soil; the air within the canopy is warmed to a lesser extent by convective sensible heat exchange. Using a sophisticated automatic feedback system to compensate for abiotic variation (ambient temperature, wind) and to maintain vegetation temperatures a set amount greater than those in a reference plot, a warming treatment can be achieved that quantitatively simulates global warming [5].

The experimental plot temperature used in the feedback control is often monitored with an IR radiometer (IRR) which has the advantage of integrating plant and soil temperature within a fairly large area (~1 m^2^). This generally works well as the main input to the feedback control, but sometimes there can be a disconnect between IRR-measured and ecologically relevant plant and soil temperatures. Although several IR heater warming experiments have been conducted or are currently underway [2, 4, 7, 8, 9, 10, 11, 12, 13, 14, 15, 16] few have monitored the specific level of warming of the plant, canopy, soil, and air microclimate. Instead the researchers make assumptions about the effectiveness of the warming system. Furthermore, the relationship between IR heater electrical power input and actual canopy warming control is complicated [4, 5, 17, 18, 19].

Two main methods for increasing the ambient CO_2_ concentration in realistic field settings are open-top chambers (OTC) and Free Air CO_2_ Enrichment (FACE). Open-top chambers are clear enclosures in which CO_2_ is enriched by injections into the chamber and rapid air circulation is employed to minimize chamber warming. Free air CO_2_ enrichment uses a system of pipes and tubing to control ambient CO_2_ in open-air settings. Some FACE systems use fans to blow pre-mixed CO_2_-enriched air across plots [20], whereas others inject pure CO_2_ at the circumference of the canopy, and rely on wind to mix and transport the CO_2_ across the experimental plots [21]. Both OTC and FACE systems have advantages and disadvantages, although FACE systems, especially ones which do not use blowers, cause fewer disturbances to the microclimate [20, 21].

Here we report data from a long-term global change experiment where warming was accomplished with an IR heater—feedback controlled system coined as “T-FACE” [4] in a factorial with Free Air CO_2_ Enrichment [21]. The goals were to characterize the soil, leaf, canopy-air, and above canopy-air warming, plus investigate potential interactions between elevated CO_2_ and warming. Although not directly warming the canopy, CO_2_ enrichment has the potential to affect the microclimate by lowering leaf conductance, thereby reducing leaf transpiration and increasing leaf and canopy temperature [14, 22, 23]. These direct effects of warming and indirect effects of CO_2_ on leaf and canopy energy balance have the potential to counter any CO_2_-induced decrease in transpiration, an important feature of ecosystem response to elevated CO_2_. Our study greatly contributes to understanding the combined effects of warming and elevated CO_2_ on canopy microclimate and aids in predicting how ecosystems will function in a warmer, CO_2_-enriched world.

## Materials and Methods

### PHACE Study Experimental Design

The “Prairie Heating And CO_2_ Enrichment” (PHACE) experiment was conducted at the U.S.D.A.-A.R.S. High Plains Grasslands Research Station, located in a semi-arid grassland in Wyoming, USA (41° 11’ N, 104° 54’ W). Vegetation at the site is a northern mixed prairie dominated (70%) by the C_4_ grass *Bouteloua gracilis* (H.B.K) Lag and C_3_ grasses *Pascopyrum smithii* (Rydb.) A. Love and *Hesperostipa comata* Trin and Rupr. The other 30% is composed of minor grass, forb and small shrub species. Basal cover by vegetation is about 50% during the peak of the growing season with 50% being bare or litter-covered soil. The soil is a fine-loamy, mixed, mesic Aridic Argiustoll. Mean air temperature is -2.5°C in January and 17.5°C in July, and the mean annual precipitation is 384 mm (132-year mean). The site is windy with summer and winter average wind speeds of 3.2 and 5.1 m s^-1^ and average gusts of 13 and 16.4 m s^-1^ during our eight year study.

The PHACE study utilized free air CO_2_ enrichment (mini-FACE) and infrared warming with two levels of CO_2_ (present-day ambient and 600ppm) and temperature (control and plus 1.5/3.0°C day/night warming) in a full factorial design with five replications. Plots were circular with a 3.3 m diameter and were hydraulically isolated with plastic water barriers buried vertically to a depth of 60 cm around the perimeter. Details of the mini-FACE CO_2_ control system, have been previously described [11, 21, 23]. The warming system is similar to that previously described [4]. Detailed description and photographs of a similar T-FACE system on paddy rice were reported [13]. Plot temperatures were increased using six 1000W heaters (model FTE-1000; Mor Electric Assoc. Inc.; Comstock Park, MI, USA) at each plot. Heaters were set 1.5 m above soil surface in a hexagonal arrangement with 2 heaters per side at an angle of 45° to horizontal [4] and pointed toward the center of the plot. Control (reference) plots had the same infrastructure as warmed plots except with un-warmed “dummy” heaters to insure similar patterns of shading and rain influence. Elevated and reference plot temperatures were monitored using IR radiometers (Model SI-111; Apogee Inst. Logan, UT, USA) mounted at 55° from horizontal at height of 50 cm from the soil surface. The field of view of the radiometers is approximately 0.5 m^2^ ground surface area. The IR radiometer was corrected for radiation emitted from the heaters and reflected from the vegetation in the 8–14 µm band [4]. Infrared temperatures in warmed and control plots were continuously measured and the differentials calculated by a datalogger (CR1000; Campbell Scientific Inc.; Logan, UT, USA). These differentials were used in a proportional integral derivative (PID) feedback loop to adjust the heater outputs via electronically controlled dimmers to maintain set temperature differences between heated and control plots of 1.5°C during daytime and 3.0°C at night [24]. The PID loop operates on IR temperatures at a frequency of one second, and average hourly temperatures were stored by the datalogger. Electricity consumption was also recorded by the datalogger to aid in project management.

The warming system was operated 24 hours per day year-round, whereas the CO_2_ elevating system was operated only during sunlight hours of the growing season, from about April 1 to November 1 each year.

### Microclimate Analyses

In 2010 we began detailed monitoring of the direct effects of the warming and mini-FACE systems on the plant and soil microclimate of the plots. This was in addition to the ongoing soil moisture and temperature measurements on all plots at the site [23]. Two replications of the full factorial of elevated CO_2_ and warming were monitored on these eight detailed plots. In addition to the IR radiometers used for warming control, temperatures were measured at five locations in the vertical profile of the plots. Thermocouples were used to monitor temperature independently from the IR technology. In each of the eight plots, a fine-wire (127µm) type E thermocouple was placed 25 cm above the soil surface to measure above-canopy temperature (the height of the vegetation was typically <25 cm). The average of two fine wire (127µm) type E thermocouples was used to measure within-canopy-air temperature at 15 cm above the soil surface. Leaf temperatures were measured on the sites’ dominant C_3_ grass species, *Pascopyrum smithii* and *Heterostipa comata* using Type T fine-wire (75 µm) thermocouples placed on the underside of two leaves of each species. Soil surface temperature (about 0.5-cm depth) was measured with a 4-probe averaging sensor (TCAV; Campbell Scientific Inc.; Logan, UT, USA). Soil temperature at 3-cm depth was measured using a Type T thermocouple. Plot air relative humidity and temperature were also measured with a RH-temperature probe (CS215: Campbell Scientific Inc.; Logan, UT, USA ) installed at a height of 15 cm. The probe was mounted in an insulated white plastic housing, which was aspirated by a small fan for two minutes prior to recording RH and temperature. All thermocouples were inspected at least once per week for integrity and for good contact with the underside of the leaves in the case of leaf temperatures. Sensors were scanned every 15 seconds and averaged for one hour time steps. IR radiometers were tested yearly and calibrated if needed at the manufacturer. The PHACE experiment operated from 2006 to 2013, but the detailed monitoring of microclimate was conducted in 2010–2012.

At the PHACE site [23], as well as many other CO_2_ enrichment studies [10, 11, 25] elevated CO_2_ leads to conservation of water by plants due to stomatal closure, resulting in higher soil water content during portions of the year. Since much of the incident radiation falls on the soil surface and water has a high specific heat, we wondered whether performance of the IR heater system, which involved warming of the combined canopy and soil, would be influenced by near surface soil water content. We therefore investigated the performance of our warming system under a range of natural and elevated-CO_2_-induced surface soil water content using June 19 to August 11, 2011 which had several strong wet-up to dry-down periods. Soil water content was measured in the 4 to 15 cm depth using 10HS probes (Decagon Devices Inc., Pullman, WA, USA).

### Statistical Methods

We performed a statistical analysis to test for significant temperature differentials due to the warming and CO_2_ treatments and their interactions (although there were differences between years, there were no CO_2_ or warming interactions with year) using PROC GLM model (SAS Inc.; Cary, NC, USA; n = 2). The contrast across CO_2_ treatment plots was to evaluate whether CO_2_-induced stomatal closure affected leaf or canopy-air temperature and whether such a response might influence the warming system. Day and night data were analyzed separately because the target warming temperature differed (1.5 vs. 3.0°C). For statistical comparisons, data were averaged over 5-hour intervals during the middle of the day (1000 to 1500 hours) and night (2300 to 400 the next day) to avoid transitional periods (sunrise/sunset). Because the main goal was to investigate plant responses, for statistical analysis the day and night intervals were averaged over the growing season: May 1 to July 31, 2010, 2011 and 2012. Also, half-hourly data were plotted for summer and winter solstice comparisons.

## Results

The CO_2_ treatment had no detectable effect on the seven environmental variables, nor did CO_2_ interact with warming to affect microclimate variables (Table 1). Therefore, subsequent data analysis of warming effects was averaged over the two CO_2_ treatments.

**Table 1:** Probabilities of warming and CO_2_ effects on microclimate temperatures and RH at the PHACE experiment.

**Day hours**	**Vegetation**		**Air**			**Soil**	
	IRR	Leaf	Canopy	Above	RH	Surface	3 cm
Warming	**0.0001**	**0.009**	0.108	0.236	0.102	**0.014**	**0.002**
CO_2_	0.997	0.316	0.283	0.648	0.286	0.591	0.466
Warming * CO_2_	0.776	0.316	0.935	0.230	0.599	0.063	0.096
**Night hours**							
	IRR	Leaf	Canopy	Above	RH	Surface	3 cm
Warming	**0.0001**	**0.0001**	**0.003**	0.290	**0.0001**	**0.0001**	**0.0001**
CO_2_	0.324	0.123	0.679	0.899	0.982	0.394	0.434
Warming*CO_2_	0.229	0.831	0.576	0.919	0.218	0.184	0.200

Data are probabilities (analysis of variance using a general linear model) from three years (2010–2012) of day and night hours over the growing season (IRR = IR radiometer; Leaf = thermocouple on underside of leaves; Canopy = air temperature within canopy; Above = air temperature above canopy; Surface = soil temperature at soil surface; 3-cm = soil temperature 3 cm below surface; RH = relative humidity above canopy). Significant P values are bolded. There were no year by treatment interactions.

**Figure 1:**
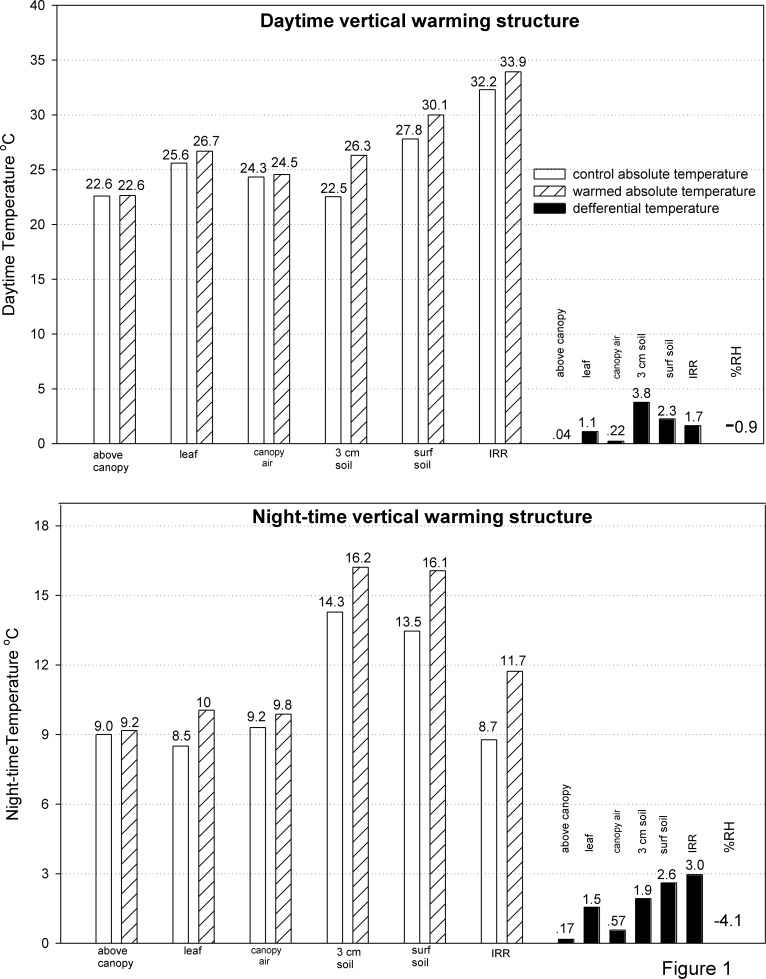
Effects of IR heaters on the vertical temperature profile in T-FACE experimental plots. Data are the mean (over three years) absolute and differential microclimate temperatures and RH of T-FACE infrared heater warmed and control plots during day and night hours (same data used in the analysis of Table 1) at the Prairie Heating and CO_2_ Enrichment experiment. IRR is ‘infrared-radiometer; all other temperatures measured by thermocouples at “above (the) canopy”, the underside of “leaf”, in the middle of the plant “canopy-air”, at “3 cm soil” and “surface” soil depths. RH is relative humidity within the plant canopy.

Both during the day and night, the IR heater system warmed the leaves, canopy-air, surface soil, and soil at 3 cm depth, as well as reducing the RH (Table 1). There was no effect of warming on the above-canopy temperature (25-cm height). During the day, differences in canopy-air temperature and RH were small (P = 0.10), but these differences were stronger at night, suggesting that the higher target temperature and calmer conditions at night (3 vs. 1.5°C) were required to detect significant differentials (Table 1; Fig. 1).

During the day, the IR heater system warmed the leaves (1.1°C) and soil (~3.0°C) quite well (Fig. 1; Table 1). The average of the differential leaf and surface soil temperature was the same as that of the IR radiometer (1.7°C) providing strong evidence that the IR radiometer measured the combination of leaf and soil temperatures in our sparse canopy plots. Note that during the day the absolute IR radiometer temperature is warmer than the individual components by several degrees, as expected due to the direct warming of the vegetation and soil surfaces by the sun.

During the night, the differential temperature of the IR radiometer was exactly on the target setting, but was higher than the average of leaf and soil temperature (3.0 vs. 2.1°C). Opposite to daytime, as expected, the IRR temperatures were cooler than the soil temperatures due to thermal radiation from the vegetation and exposed soil surfaces to the cold night sky. Absolute relative humidity was reduced in the warmed plots by 4% at night, but by only 1% during daytime (Fig. 1).

**Figure 2:**
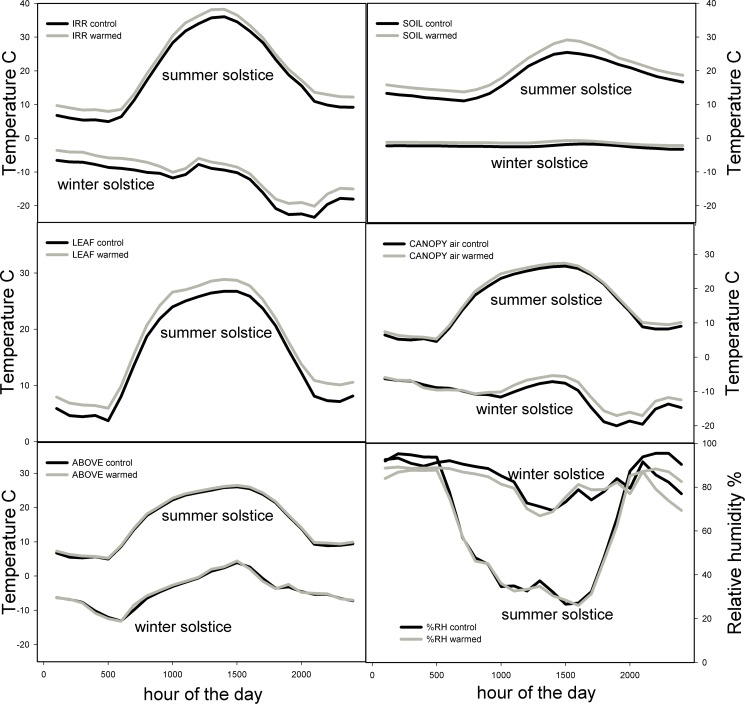
Effects of IR heaters on daily patterns of the vertical temperature profile in T-FACE experimental plots. Graphs show the diurnal patterns of five T-FACE microclimate temperatures and canopy relative humidity (15-minute averages) on the summer and winter solstice of 2011 at the Prairie Heating and CO_2_ Enrichment experiment. Gray lines are the IR heater-warmed plots, black are the reference-control plots. The day target warming was 1.5°C and night 3.0°C. There were no LEAF thermocouples in the winter.

Overall, the warming system performed equally well in the winter and summer, especially based on the IR radiometer data (Fig. 2). Surface and 3-cm soil temperatures were less warmed in the winter, likely due to frozen soil. Transitional periods from winter to spring and autumn to winter might be strongly affected by warming, potentially promoting and extending the period of unfrozen soil and soil biota activity. Note that the shape of the leaf temperature curve closely tracked that of the IR radiometer showing that leaf warming was tightly linked to the warming system (there were no thermocouples on leaves during the winter). As shown in Fig. 2 the plant canopy-air was slightly warmed by the IR heaters and the above canopy-air not warmed.

**Figure 3:**
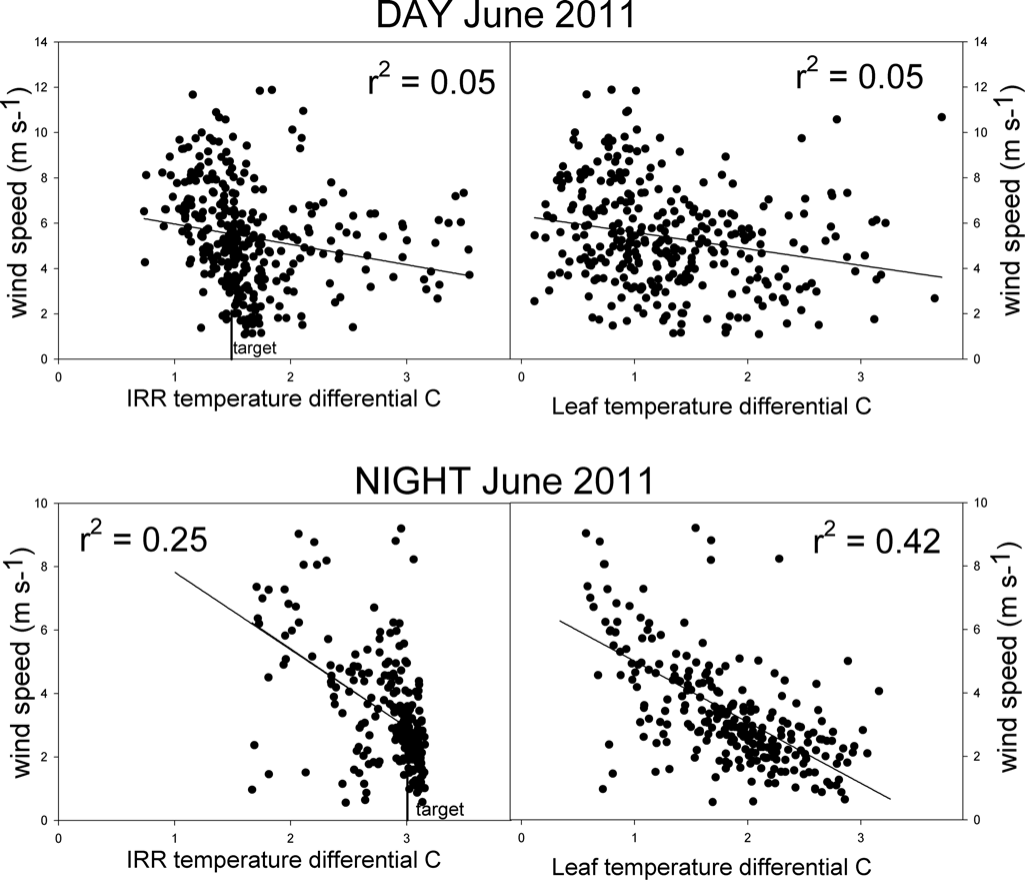
Effects of wind on performance of IR warming in T-FACE experimental plots. Graphs show hourly wind speed vs. infra-red radiometer and leaf temperature differentials (warmed minus control) of T-FACE plots during the DAY and NIGHT (1.5 and 3.0°C target differentials) during June of 2011 at the Prairie Heating and CO_2_ Enrichment experiment.

We were initially concerned that controlling the warming treatment at our windy Wyoming site would be problematic. However, wind speeds up to 12 m s^-1^ had only small effects on warming performance (Fig. 3). Both day and night, the IR radiometer differentials are clustered near the target set point warming differentials, mostly regardless of wind speed. At night wind speed was negatively correlated with leaf temperature differentials (r^2^ = 0.42***) and the IR radiometer differentials (r^2^ = 0.25**). Due to the 3.0°C target warming, the power demand on the warming system was higher at night and appears to have been somewhat less effective during high winds. Leaf temperature would be most susceptible to strong winds. Table 2 shows the correlation coefficients of wind vs. the other temperature variables which were all non-significant.

**Table 2:** The relationship of wind speed with microclimate temperature at the PHACE experiment.

	**IRR**	**Leaf**	**3cm soil**	**Surface soil**	**Canopy-air**	**Above canopy**	**RH**
Day wind speed	0.05 (ns)	0.05 (ns)	0.005 (ns)	0.02 (ns)	0.07 (ns)	0.09 (ns)	0.004 (ns)
Night wind speed	0.25 **	0.42 ***	0.05 (ns)	0.02 (ns)	0.19 (ns)	0.1 (ns)	0.12 (ns)

Values are correlation coefficients (r^2^) and probabilities (** = <0.01; *** = <0.001; analysis of variance using general linear models) of day and night hourly average wind speed with microclimate measurements in June 2011 (IRR = IR radiometer; Leaf = thermocouple on underside of leaves; 3-cm soil = temperature 3 cm below the soil surface; Surface soil = temperature at soil surface; Canopy-air = temperature within canopy; Above canopy = air temperature above canopy; RH = relative humidity above canopy).

There were no significant relationships between upper depth soil water content and the seven microclimate variables (data not shown). The IR heater system operated very well over a range of soil moisture levels.

**Figure 4:**
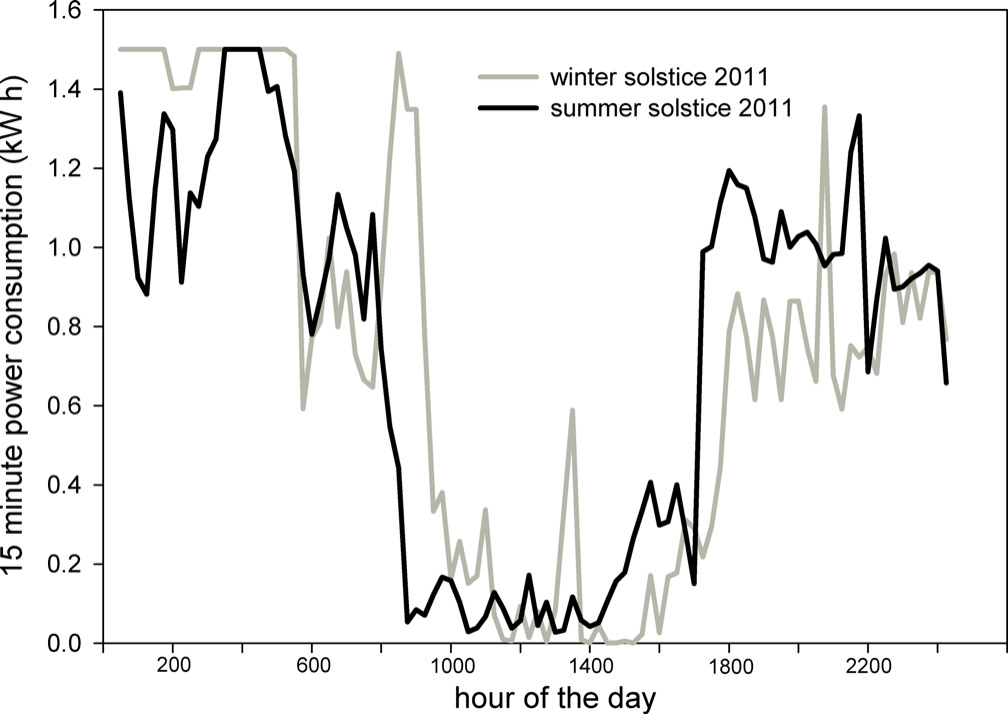
Daily variation in power use in an IR warmed, T-FACE experimental plot. Power consumption during 15 minute periods for one 6-IR-heater T-FACE plot during the 24 hours on the summer and winter solstice of 2011 at the Prairie Heating and CO_2_ Enrichment experiment. Flat lines near 1.5 kWh show maximum electricity potential.

During the same three month summer period used for temperature averaging, the power consumption for a single six IR-heater plot during daytime was 2528 kWh and at night was 2382 kWh. The night period was only about 9 hours long, but the target warming (3.0°C) was double the day target (1.5°C). During the three months of December, January and February (2011 to 2012) the day power consumption was 1400 kWh and night was 3460. The long night period and warmer target temperature resulted in most of the power needs occurring at night. The local price for electricity was about $0.1 per kWh, and therefore, the annual energy cost was about $2,000 per plot (7.1 m^2^). Cost for liquid CO_2_ were also about $2000/year/plot (plus significant tank rental fees). Fig. 4 shows power consumption for one PHACE plot for summer and winter solstice of 2011. Power required to maintain the set temperatures varied greatly during single day and night periods. Periods of greatly changing power demand correspond with day/night warming targets and variable wind speed. Average daily power consumption for one plot was about 53 kWh in both summer and winter.

## Discussion

Air, plant and soil temperature are critical drivers of many ecological functions; transpiration, evaporation, plant physiological and soil microbial activity to name a few. Our study is unique among IR warming experiments in monitoring the temperature of many ecologically important locations under IR warming (Table 3). Most (8 of 10) previous studies did indeed monitor soil temperature, but most did not monitor canopy temperature (1 of 10; with the exception of using the IR radiometer for control), leaf temperature (2 of 10), or surrounding air temperature (2 of 10). Even though most studies measured soil temperature, some did only daily spot checks which does not characterize diurnal variation. This is especially important in passive-constant output IR heaters with no feedback control as day vs night efficiency of warming is vastly different [7, 9, 25]. The constant output IR warming systems sometimes warmed very little during the day and typically more at night. This information is essential for interpreting ecosystem responses [9, 11].

**Table 3:** Published field infrared heater warming studies.

**Reference**	**ecosystem**	**IRR heating method**	**Elevated CO_2_?**	**canopy Temperature method/result**	**soil Temperature method/result**	**leaf Temperature method/result**	**air Temperature method/result**
Harte et al. 1995	montane meadow	Passive	no	not measured	TC @5–25cm 0.6 avg.	not measured	not measured
Loik et al. 2000	Montane meadow	Passive	No	not measured	TC @ 5, 12cm 2 at noon	TC under leaf 0°C	not measured
Wan et al. 2002	Prairie	Passive	no	not measured	TC @ 2.5cm 2 average	not measured	TC @ 25cm 0 D/2 N
Dukes et al. 2005	annual grassland	Passive	yes	not given 1°C	not measured	not measured	not measured
Hovenden et al. 2006	perennial grassland	Passive	yes	not measured	TC @ 1cm 0.8 avg.	TC 2.1	Hygrometer @ 5cm 2.16
Xia et al. 2009	Mongolian steppe	Passive	no	not measured	TC @ 10cm 0.17 D/0.38 N	not measured	not measured
Luo et al. 2010	Tibetan plateau	Feedback controlled	no	IRR 1.2D/1.7N	TC @ 5–20cm 1.6 to 0.5 avg.	not measured	not measured
Rehmani et al. 2011	rice crop	Feedback controlled	no	IRR 1.3D/2.7N	not measured	not measured	not measured
Wall et al. 2011	Wheat crop	Feedback controlled	yes	IRR 1.3D/2.7N	TC @ 10cm 1.3 avg.	not measured	not measured
Gaihre et al. 2014	rice crop	Feedback controlled	no	IRR 1.1D/2.6N	TC @ 5cm 0.4 avg.	not measured	not measured
Current study	mixed grassland	Feedback controlled	yes	IRR & TC @ 15cm 1.7D/3.0N 0.2D/0.57N	TC @ surf & 3cm 2.3D/2.6N 3.8D/1.9N	TC under leaves 1.1D/1.5N	TC @ 25cm.04D/.17N

Warming method Passive: not feed-back controlled, continuous output.

FBC: Feed-back controlled, differential from reference output.

TC = thermocouple

IRR = infra-red radiometer

D = day, N = night.

Results are differential temperature between warmed and control plots in °C.

The T-FACE ecosystem warming system operated year round at the severe weather Wyoming site with few problems. Occasionally an IR heater would fail but these were simple to replace. Users should monitor system performance at least weekly, and have backup heaters ready to install. The reference—control feedback system worked extremely well during the day with a 1.5°C target differential. Warming of plants and soil was slightly less than desired at night even though the IR radiometer precisely controlled to its target (3.0°C) confirming the importance of independent monitoring. The feedback controlled system warmed much more consistently than the passive IR heater systems used at some sites [7, 9].

Although warming decreased soil water content by an average of 13.1% [23], there was no influence of soil moisture in the 4–15 cm depth range on the performance of our warming system. The warming control system adjusted well for effects from variations in soil moisture, whether from precipitation or induced by the CO_2_ treatment. It is likely that IR warming will periodically be affected by soil moisture conditions such as faster snow melt but this scenario should be minor in a long-term study with the exception of locales dominated by snow.

Relative humidity in the plant canopy was reduced under warming, but by only 1% in daytime and 4% (absolute) at night. These small changes in RH occurred because canopy-air temperature was only slightly affected by IR warming, which heated mostly the leaf and soil surfaces (Fig. 1). This is good since RH is not expected to be much affected by global climate change [1], and significant warming-induced reductions in RH could increase canopy evapo-transpiration by increasing the canopy to air vapor pressure deficit. The minor change in daytime RH from IR warming had little effect on vapor pressure deficit and ET in our experiment. This consequence of IR warming has been well discussed in the literature [4, 24, 6]. Although not implemented in this experiment, a supplemental irrigation system was proposed [4, 24] that would adjust for this artifact of IR warming systems.

We expected that the elevated CO_2_ treatment would warm leaf temperatures during the day (CO_2_ is not elevated at night) due to partial stomatal closure and lower transpirational cooling, as has been documented in cropped systems [22]. Reductions in leaf stomatal conductance from exposure to CO_2_-enriched atmospheres are commonly reported in the literature [14, 25, 26, 27]. However, the consequences of such stomatal closure for transpiration and resulting soil water content at the canopy level continue to be debated due to the off-setting effects of higher leaf area of CO_2_-enriched canopies [28, 29, 30], and an increased vapor pressure deficit which develops when stomatal closure leads to higher leaf temperature [28, 31]. Both would tend to increase transpiration and reduce the water conservation effect of elevated CO_2_. We believe the positive effect elevated CO_2_ has on primary productivity in semi-arid grasslands of the western Great Plains [23, 32] is mostly a direct result of water savings resulting from partial stomatal closure. However, this reduction in canopy conductance was not strong enough to increase leaf temperatures at the PHACE site.

We previously reported that a relatively small increase in leaf area under CO_2_-enriched conditions was insufficient to overcome the leaf and canopy level water conservation attributed to CO_2_-induced stomatal closure in a Wyoming mixed-grass prairie [23]. We suspect that the narrow leaves and small leaf area of this grassland canopy in combination with windy conditions minimized changes in leaf temperature due to stomatal closure [33], which provides further support for the consistent effects of CO_2_ on plant and soil water relations in dry grasslands of this region [26, 32, 34].

Our group earlier reported [23] that the IR radiometer temperature was within 0.5°C of the daytime target temperature 69% of the time, and night time target 72% of the time using 2007, 2008 and 2009 data. IR radiometer data from years 2010, 2011 and 2012 showed a small improvement in this year-round reliability of the warming system (75% both day and night; data not shown). These results are comparable to similar T-FACE system installed in China, where target day and night differentials (1.3. and 2.7°C) were maintained within 0.5°C, 67% of the time [13].

## Conclusions

At our severe weather Wyoming, USA, site, the T-FACE plot warming system proved to be a robust and precise method for year-round experimental warming of plants and soils. The IR radiometer proved an excellent control input method (especially during the day) and has the advantage of integrating over a large surface area. The desired leaf and soil warming were achieved with only small humidity concerns. The mini-FACE CO_2_ enrichment system had no significant direct or interactive effects with warming on microclimate attributes. Multi-factor global change studies are challenging and expensive to conduct, and are still quite rare. Our PHACE experiment utilized the current best and non-intrusive methods for field study resulting in treatment precision that gives confidence to the many plant and soil ecosystem response measurements. Also, our results suggest that passive, non-feedback controlled warming systems are likely poor at simulating predicted global warming temperatures. Electricity (and CO_2_) costs are a major factor in experimental design. Existing and subsequent studies conducted in sparse canopy, semi-arid ecosystems can look to our results for pertinent guidelines. However, our results demonstrate the necessity of monitoring the microclimate in future warming studies.
